# Photo, thermal and chemical degradation of riboflavin

**DOI:** 10.3762/bjoc.10.208

**Published:** 2014-08-26

**Authors:** Muhammad Ali Sheraz, Sadia Hafeez Kazi, Sofia Ahmed, Zubair Anwar, Iqbal Ahmad

**Affiliations:** 1 Baqai Institute of Pharmaceutical Sciences, Baqai Medical University, 51, Deh Tor, Toll Plaza, Super Highway, Gadap Road, Karachi 74600, Pakistan

**Keywords:** chemical degradation, degradation products, photodegradation, riboflavin, stability, thermal degradation

## Abstract

Riboflavin (RF), also known as vitamin B_2_, belongs to the class of water-soluble vitamins and is widely present in a variety of food products. It is sensitive to light and high temperature, and therefore, needs a consideration of these factors for its stability in food products and pharmaceutical preparations. A number of other factors have also been identified that affect the stability of RF. These factors include radiation source, its intensity and wavelength, pH, presence of oxygen, buffer concentration and ionic strength, solvent polarity and viscosity, and use of stabilizers and complexing agents. A detailed review of the literature in this field has been made and all those factors that affect the photo, thermal and chemical degradation of RF have been discussed. RF undergoes degradation through several mechanisms and an understanding of the mode of photo- and thermal degradation of RF may help in the stabilization of the vitamin. A general scheme for the photodegradation of RF is presented.

## Review

The study of photo, thermal and chemical degradation in the stability of drugs is one of the most concerned areas in the field of drug development and formulation. In British Pharmacopoeia [1] a number of drugs have been mentioned which require protection from light or need to be stored at a specific temperature. The consequences of exposure of such drugs to light or heat may result in the loss of potency and formation of degradation products which could be harmful to the human body. However, not every drug shows similar behavior when exposed to unfavorable conditions, e.g., nifedepine [2] and cyanocobalamin [3] degrade rapidly on exposure to light whereas ephedrine [4] shows a slower rate of photodegradation. Information regarding the stability and degradation of a particular drug is pharmaceutically significant in the determination of its therapeutic outcomes, adverse effects, handling, packaging and labeling protocols, etc. [5–9]. The most common approach to cope with the problem of photosensitivity is the use of amber colored bottles or light resistant packaging. Thermal sensitivity can be dealt by manufacturing and storing the drug under controlled temperature conditions. Similarly, the chemical degradation of the drug may be controlled by changes in pH, buffer, solvent composition, exclusion of air and use of stabilizer. In case if this is not the appropriate solution then modification of a formulation can be considered to improve the stability and shelf-life of the product.

Riboflavin (RF) was discovered as a yellow green fluorescent compound and was isolated from a yellow enzyme [10]. It is present in almost all green, leafy, rapid growing vegetables where it is bound to proteins. Whereas dairy products, meats, fruits etc. also contain RF in considerable amounts and it is present in all natural unprocessed foods in various amounts [11–12]. RF takes part in several electron transfer processes and is known to transfer single electrons, hydrogen atoms and hydride ions to a substrate. In this way it may contribute in redox reactions as either a one- or two-electron mediator thus proving itself as a necessary molecule for the flavin-dependent enzymatic reactions in biological systems. The two major coenzymes, flavin mononucleotide (FMN) and flavin adenine dinucleotide (FAD), account for the vitamin activity in human nutrition [13].

**Figure 1:**
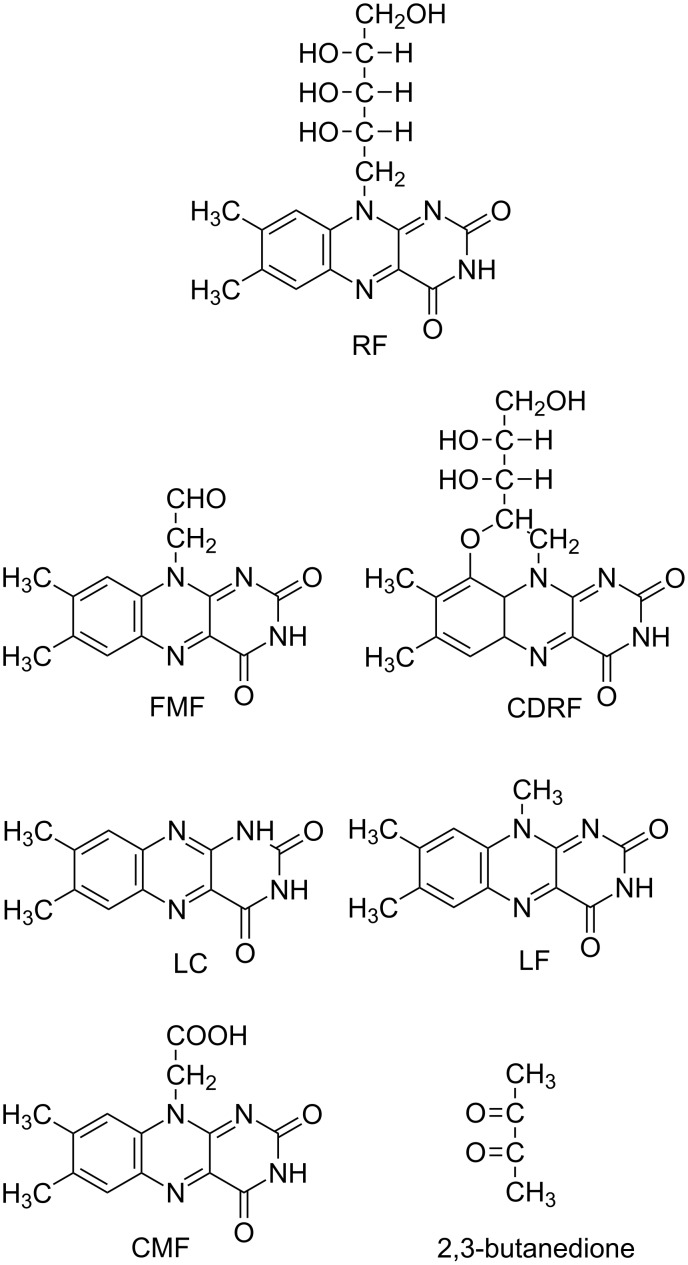
Structures of RF and its photoproducts.

RF is among the most widely studied compounds in terms of photostability and degradation in aqueous and organic solvents. It shows strong absorption at 223, 267, 373 and 444 nm in the UV and visible regions in aqueous solution and is degraded into various photoproducts on exposure to light [1]. These products include formylmethylflavin (FMF), lumichrome (LC), lumiflavin (LF), carboxymethylflavin (CMF), 2,3-butanedione, a β-keto acid and a diketo compound [14–33]. Some of the products reported earlier [14,20,34] still need to be identified. In the presence of divalent anions, such as phosphate (HPO_4_^2–^) and sulfate (SO_4_^2–^), the photodegradation of RF leads to the formation of cyclodehydroriboflavin (CDRF) [21,30,35]. The structures of RF and its photoproducts are shown in Figure 1.

FMF is an intermediate in the photodegradation of RF and is more sensitive to light than RF [15–17,27,32–33]. It is hydrolyzed to LC and LF [18–19,22–33] and is oxidized to CMF [20,27,32]. Both LC and LF are also sensitive to light, with LC having relatively better stability [36]. The nature of the photoproducts of RF depends on the reaction conditions such as solvent, pH, buffer kind and concentration, oxygen content, light intensity and wavelengths.

### Photochemical reactions of riboflavin

A number of reviews have been published on the mechanisms involved in the photochemical reactions of flavins [18,37–49]. Both excited singlet and excited triplet states of RF are implicated in the photodegradation reactions by different mechanisms [36,41–42,46–47,50–57].

**Figure 2:**
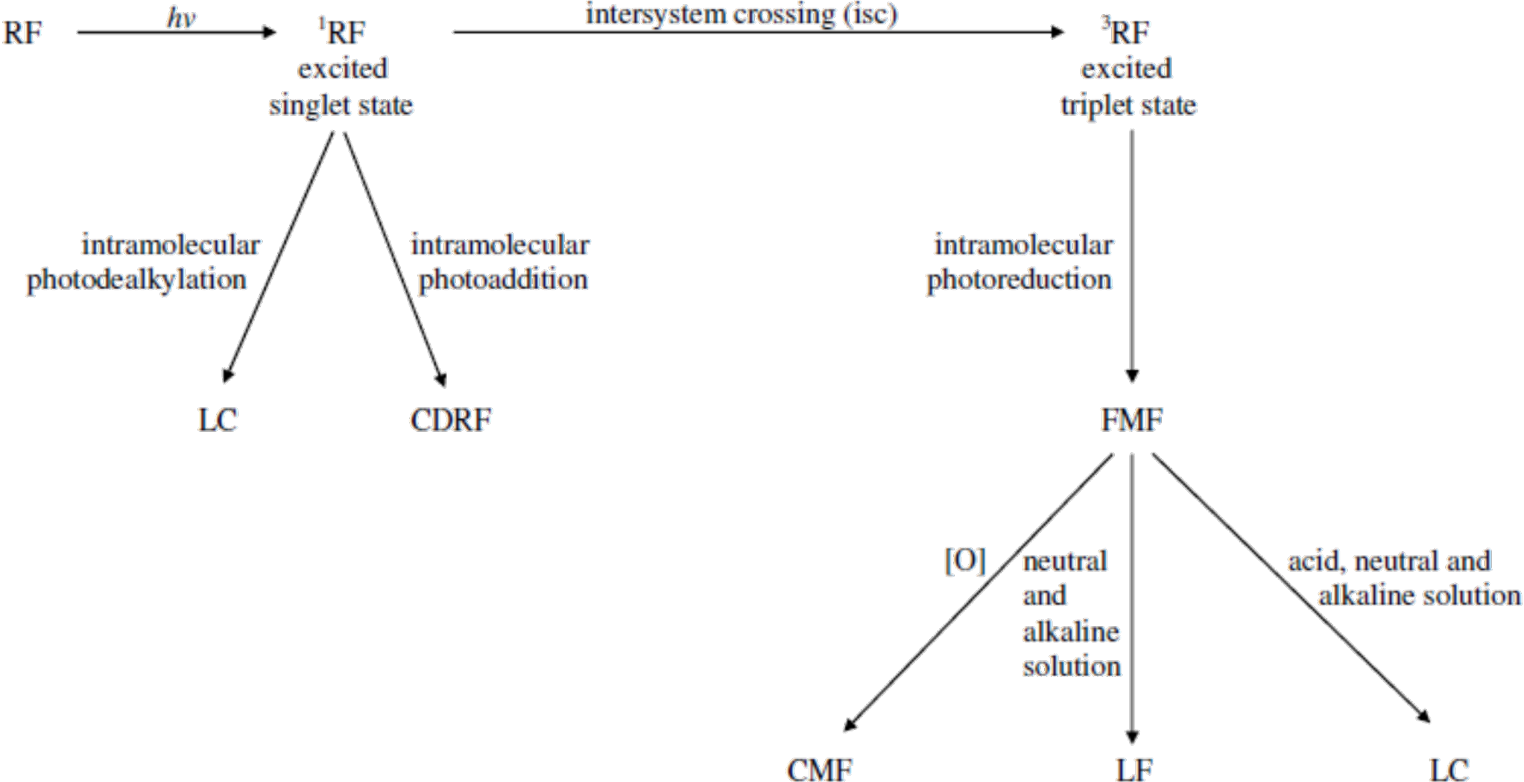
A general scheme for the photodegradation of RF in aqueous solution.

RF also forms singlet oxygen from the ordinary triplet oxygen under light by the excited triplet RF and triplet oxygen annihilation mechanism which plays a part in photosensitized reactions [58–59]. FMF, LC and LF are formed by the excited triplet state of RF [24,36,59] whereas the excited singlet state plays a role in the formation of LC and CDRF [14,24,35,42]. The excitation of the RF molecule on the absorption of light takes place very rapidly as the life spans of flavin excited singlet and triplet states are approximately 5 ns [60] and 1 ms [61], respectively. The reactions involved in the photochemical degradation of RF include photoreduction, photoaddition and photodealkylation. These reactions may occur intramolecularly or intermolecularly or often simultaneously [18,23,25,30,46–47,49,62]. However, a clear distinction between these reactions in the photodegradation of RF lacks information. A general scheme for the photodegradation of RF in aqueous solution is presented in Figure 2.

### Factors affecting photodegradation of riboflavin

The photochemical reactions involved in the degradation of RF are affected by a number of factors that are discussed as follows:

#### Radiation source, intensity and wavelengths

The emission characteristics of the radiation source are an important factor that plays a significant role in the photodegradation of RF. Around 30% of RF is destroyed by sunlight in milk within only 30 minutes of exposure [63]. In the dark, RF is stable and remains unchanged under specified conditions for prolonged periods of time [11,36,64]. In the dry form, RF is not much affected by light while in the solution form it is rapidly degraded to various photoproducts through a variety of reactions under aerobic and anaerobic conditions [11,14–15,18,20–21,23–35,65].

A number of studies have been conducted by employing different low and high intensity radiation sources emitting at different wavelengths in the UV and visible regions for the photolysis of RF [24,26,34,66–71]. A comparison between UV and visible radiation sources for the photodegradation of RF has been made by Ahmad et al. [24,26]. Similar photoproducts were formed when the aqueous solutions of RF were exposed to either of the radiation source, however, the rate of reaction was higher on UV irradiation as compared to that of the visible irradiation. Similarly, the magnitude of the formation of the photoproducts was higher in solutions exposed to UV light as compared to the visible light. The difference in rates might be due to the higher intensity of the UV radiation (medium pressure mercury vapor lamp, 125 W), i.e., 2.19 ± 0.12 × 10^18^ quanta s^−1^ as compared to the visible sources (high pressure mercury vapor fluorescent lamp, 125 W and tungsten lamp, 150 W), i.e., 1.14 ± 0.10 × 10^17^ and 1.06 ± 0.11 × 10^16^ quanta s^−1^, respectively [24,26]. Previously it was reported by Sattar et al. [72] that the wavelengths in the range of 350–520 nm are damaging to RF solution especially in the range of 415–455 nm [11,72]. However, the method of analysis performed by these workers was not selective as that of Ahmad and Rapson [34] and Ahmad et al. [24,26], and therefore, an accurate determination of the vitamin content may not have been made to assess the rate of degradation.

In another study performed on RF tablets, the samples were exposed to three different irradiances of 250, 550 and 765 W/m^2^ of xenon lamp emitting in the wavelength range of 300–800 nm. The highest color change in the tablets from yellow to green was observed at an irradiance of 250 W/m^2^ at a dose of <900 kJ/m^2^ after initial exposure. None of the excipients of the tablets had the green color nor became green after light exposure. The discoloration of RF tablets was due to the action of visible (blue) light (i.e., >400 nm). LC was identified as the only degradation product in the samples [73]. The various radiation sources used for photodegradation studies of drugs have been discussed by Moore [74].

#### Effect of pH

The photodegradation of RF is greatly affected by the pH of the medium and the photoproducts thus obtained are also dependent on pH. The main photoproducts of RF are FMF and LC which are formed at pH 1–12 and LF at pH 7–12 due to the oxidation of the ribityl side-chain. Along with these major photoproducts some minor products are also formed such as CMF at pH 1–12, and a β-keto acid and a diketo compound at around pH 10–12. The latter two photoproducts are formed by the isoalloxazine ring cleavage on alkaline hydrolysis of RF [20,24,32,34,75–77]. The pH of the solution has a significant effect on the photostability of RF. Under acidic and neutral pH conditions, RF is photodegraded to LC whereas in alkaline media it forms LC along with LF. Both these major photoproducts are formed via the triplet excited state through the mediation of FMF, which serves as an intermediate in the photolysis of RF [10–11,18,22–36,59,78]. LC and LF are non-volatile and are biologically inactive [10–11,59]. They also degrade under light once they reach their respective maximum concentrations at various pH values. However, LC is more stable at lower pH than at a higher pH [36], probably due to its protonation. LF is further degraded in the alkaline solution in the pH range of 14–14.6 at room temperature and forms anionic 7,8-dimethylisoalloxazine, anionic methylisoalloxazine, and quinoxaline derivatives of 1,2-dihydro-2-keto-1,6,7-trimethylquinoxaline-3-carboxylic acid, 2-methoxy-6,7-dimethylquinoxaline-3-carboxylic acid, methylquinoxaline-2-ol and 3-hydroxy-1,6,7-trimethyl-1*H*-quinoxaline-2-one by isoalloxazine ring cleavage [79]. A volatile compound with buttery odor has also been detected in RF solutions after prolonged light exposures in 0.1 M phosphate buffer at different pH values (4.5, 6.5 and 8.5). This compound has been identified as 2,3-butanedione and is produced from the side-chain of RF by the action of singlet oxygen. Its formation is greatly affected by the pH of the medium as the highest content of this compound was found at pH 6.5, followed by 4.5 and 8.5 [59].

**Figure 3:**
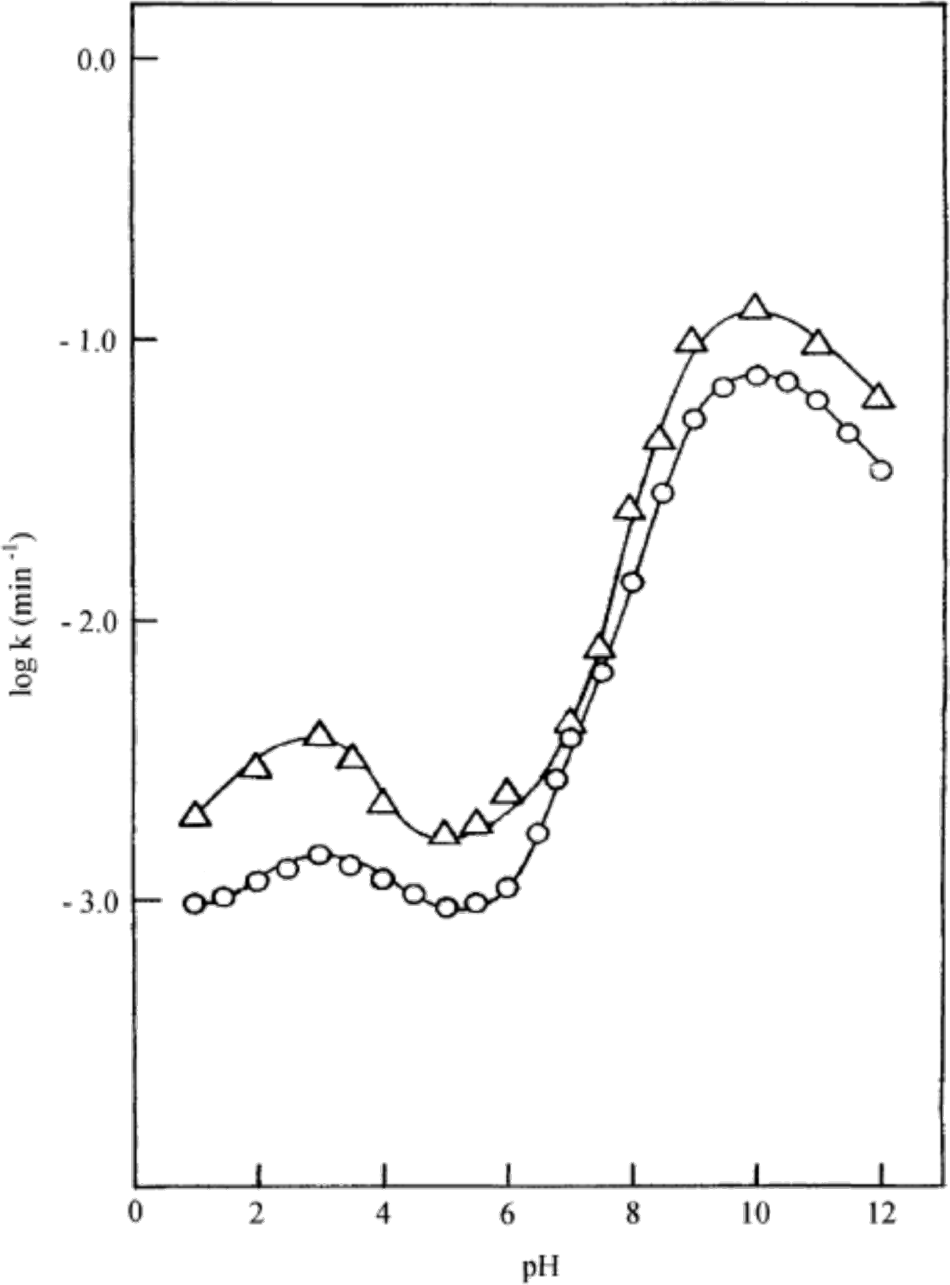
log *k*–pH profiles for the photolysis of RF in aqueous solution using UV light (∆) and visible light (**○**). UV lamp emission at 313 and 366 nm (125 W medium pressure mercury vapor lamp, 2.19 ± 0.12 × 10^18^ quanta s^−1^), Visible lamp emission at 405 and 435 nm (Philips HPL N 125 W high pressure mercury vapor fluorescent lamp, 1.14 ± 0.10 × 10^17^ quanta s^−1^). Reproduced with permission from [24]. Copyright 2004 Elsevier.

RF is highly sensitive to pH and has p*K*_a_ values of 1.7 and 10.2 [78]. The rate of photolysis of RF depends on its ionization states and their susceptibility to excitation. Ahmad et al. [24] studied the kinetics of RF over a wide pH range of 1–12 and evaluated its effect on the rate of photolysis. They determined the optimum range for the stability of RF aqueous solutions around pH 5–6 due to its lower redox potentials in this region (Figure 3).

**Figure 4:**
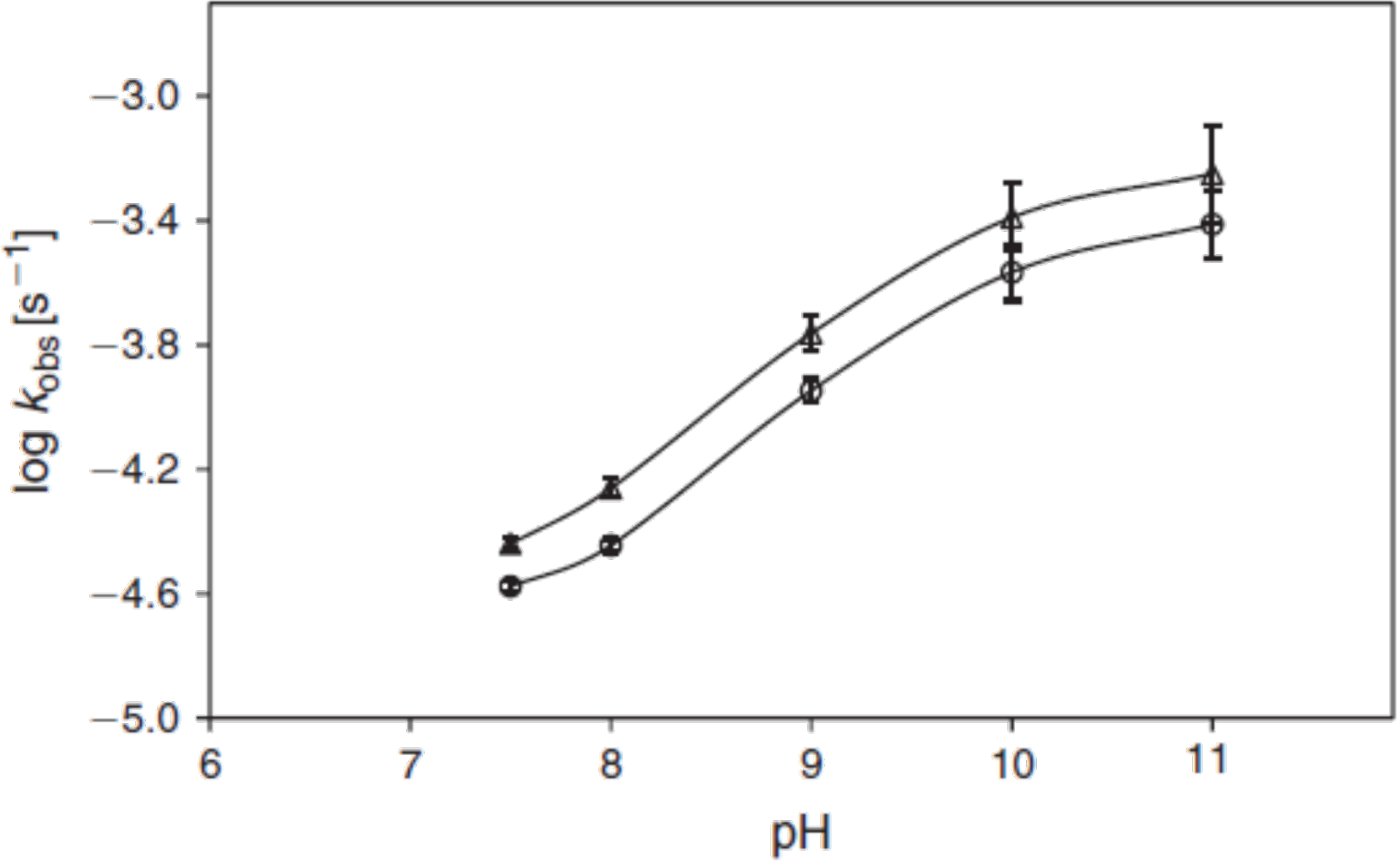
log *k*–pH profiles for the photolysis of FMF (10^−4^ M) in alkaline solution under aerobic (**○**) and anaerobic (∆) conditions irradiated for 1 h at 25 ± 1 °C using a Philips 25 W fluorescent lamp (emission at 405 and 435 nm, intensity 4.52 ± 0.15 × 10^16^ quanta s^−1^). Reproduced with permission from [32]. Copyright 2013 CSIRO Publishing.

**Figure 5:**
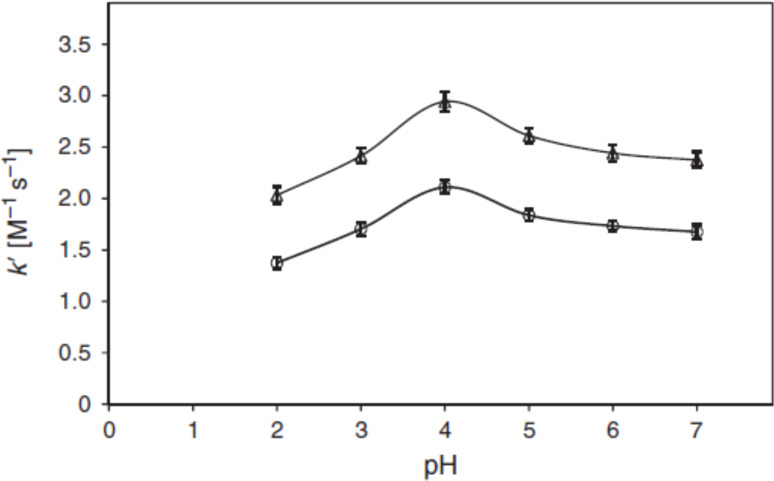
*k'*–pH profiles for the photolysis of FMF (10^−4^ M) in acidic solution under aerobic (**○**) and anaerobic (∆) conditions. Experimental conditions are the same as in Figure 4. Reproduced with permission from [32]. Copyright 2013 CSIRO Publishing.

It was also observed that the rate of photolysis of RF follows apparent first-order kinetics and is slowest in the pH range of 5–6 and is then increased tremendously (about 80 folds) in the alkaline region reaching a maximum at pH 10. This is probably due to the higher reactivity of the flavin triplet in this region [24] (Figure 3). The slight decline above pH 10 is due to the anion formation (p*K*_a_ 10.2). In acidic region, the slight increase (about 2 folds) in the rate of degradation of RF at pH 3 is due to the involvement of two pathways causing direct formation of LC (through excited singlet state) as well as through FMF (by excited triplet state) where the dominant role is played by the excited singlet state. Such formation of LC has also been reported by Song and Metzler [80] and Cairns and Metzler [14]. The non-ionized forms of RF are more susceptible to photodegradation as compared to the ionized forms and the optimum pH range for maintaining the vitamin preparations is 5–6 [24]. On the contrary, the kinetic study for the photolysis of FMF in the pH range 2.0–11.0 indicated two different orders of reactions. Its photolysis in alkaline medium (pH 7.5–11.0) takes place by first-order kinetics (Figure 4) and in acidic medium (pH 2.0–7.0) it follows second-order kinetics (Figure 5) with the maximum rates at around pH 11.0 and 4.0, respectively [32]. The effect of pH on the photodegradation of RF is a vastly studied parameter and its effect with respect to temperature, buffers and complexing agents will be discussed in the later sections.

#### Effect of anaerobic environment

The anaerobic degradation of RF in the presence of light has been studied by various scientists [15,34,38,80–81]. Anaerobic photobleaching of RF in the absence of an added electron donor has been investigated by Holmström and Oster [81]. The anaerobic irradiation of RF causes intramolecular photoreduction of the isoalloxazine ring which leads to the fading of yellow color. However, if air is introduced into the partially irradiated solutions, the yellow color of RF may return to some extent due to the reoxidation of the isoalloxazine ring. The amount of color restored depends on the time of irradiation as little color will return with more photoreduction due to photodegradation of RF [15,81–82]. Anaerobic photodegradation of RF has also been studied in various alcohols and alcohol/water mixtures alone [83] or with a water-soluble analog of vitamin E, namely trolox [84].

A photolysis study on four derivatives of RF performed in methanolic solutions identified a more efficient photodegradation in anaerobic environment rather than in the presence of oxygen for two of the derivatives, i.e., 5-deaza-RF and iso-6,7-RF. Whereas no significant influence of oxygen was noted on the photolysis of 3-benzyl-LF. The fourth derivative 3-methyl-tetraacetyl-RF, was found to be more photostable than RF. The excited triplet state was found to be involved in the photodegradation of the ribityl side chain [85]. In a kinetic study performed on the photodegradation of FMF in phosphate buffer at pH 2.0–11.0, higher rates were observed for the solutions irradiated under anaerobic conditions as compared to those exposed under aerobic conditions. The higher rates in anaerobic environment might be due to the existence of a greater number of excited singlet states of flavins compared to that of the aerobic environment as a result of singlet quenching by oxygen [32].

#### Effect of buffers

**Figure 6:**
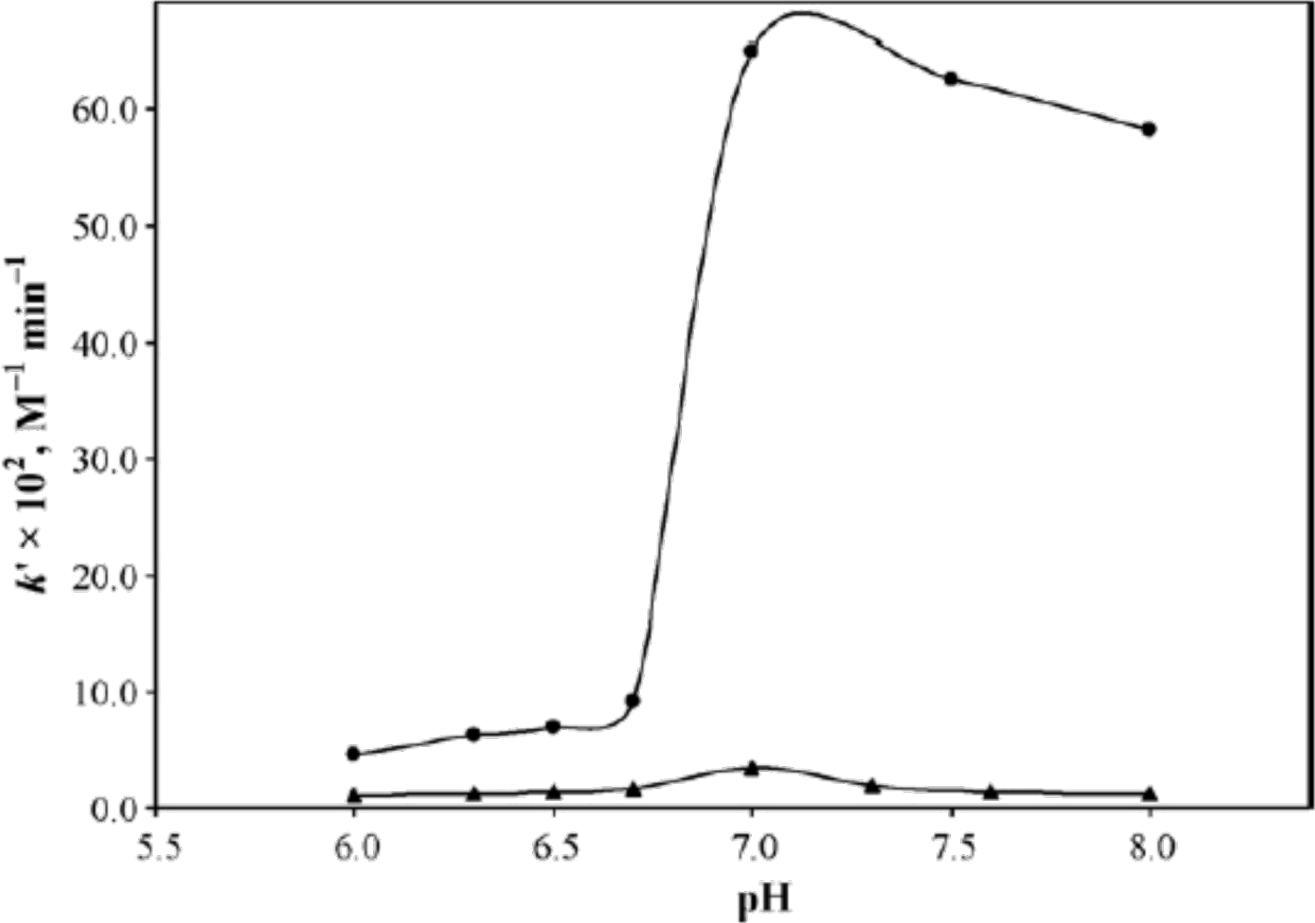
Plots of *k*` versus pH for phosphate (▲) and sulfate (●) anion-catalyzed photodegradation of RF (5 × 10^−5^ M) at 25 ± 1 °C using a Philips HPL N 125 W high pressure mercury vapor fluorescent lamp (emission at 405 and 435 nm, intensity 1.15 ± 0.10 × 10^17^ quanta s^−1^). Reproduced with permission from [30]. Copyright 2010 Elsevier.

Buffers, their concentration and ionic strength have shown to play an important role in the photodegradation of RF in aqueous solution. Different studies have shown the catalytic effect of buffer species including phosphate, sulfate, acetate and carbonate on the RF solutions [21,23,25–26,30,35,38,86–88] while borate and citrate have produced a stabilizing effect [28,31]. Solutions containing divalent anions have the tendency to catalyze the photodegradation of drug substances by break down the activated complex [89]. Similar effects have been observed for RF when its solutions were irradiated in the presence of different divalent anions such as hydrogen phosphate and sulfate (buffered solutions), tartrate, succinate and malonate (unbuffered solutions). These anions changed the mode of photodegradation of RF and caused intramolecular photoaddition via the RF-divalent anion complex formation along with the normal photolysis (intramolecular photoreduction) at pH values of 7.0 [21,23,25–26,30] or 6.0–8.0 [30,35] and hence lead to the formation of CDRF. The RF complexes formed with the unbuffered anions were comparatively weaker in their catalytic activity than those of the buffered complexes. This was also evident from the fluorescence data that showed lower fluorescence quenching by unbuffered anions as compared to that of phosphate species [30]. A much faster rate of RF photodegradation has been observed for sulfate anions than for phosphate anions (Figure 6) due to a strong complex formation, better electronegative character and the existence of a greater amount of these anions (100%) than the phosphate anions (38%) at pH 7.0 in 1.0 M solutions [21,30].

Thus, phosphate and sulfate anions show some differences in their mode of action which was evident from the rate of formation of CDRF and LC in their presence. This was also supported by the fluorescence quenching of the two anions which were almost similar at pH 7.0 from 0.2–1.0 M. In spite of faster rate, the ratios of CDRF/LC were higher for phosphate (0.74) than for sulfate (0.48) suggesting an increased formation of LC directly from the excited singlet state in the presence of sulfate anions [30]. The photoaddition reaction involved in the degradation of RF has been found to be further enhanced in the presence of caffeine which results in a further decrease of the fluorescence of RF in phosphate buffer [35]. The phosphate anions have also been found to catalyze the photolysis of FMF at pH 7.0 [32] and 2,3-butanedione [59] at various pH values. It is interesting to note that the formation of 2,3-butanedione is not dependent on the presence of phosphate buffer as it was also produced in purified water after light exposure. However, the presence of phosphate species has been found to accelerate its formation. It is more dependent on RF concentration as it was observed that lower RF contents induced slower 2,3-butanedione formation [59]. An increase in RF photodegradation was also observed when its solution was irradiated by visible light with a herbicide, 2,4-dichlorophenoxyacetic acid, in the presence of Britton–Robinson buffer at pH 6 [90]. Similarly, a catalytic effect has also been noted in acetate and carbonate buffers for RF where a change in rate of degradation was observed with an increase in pH [88].

The concentration of buffer anions has been shown to affect the photodegradation of RF. An increase in the rate of photodegradation of RF has been found with increasing ionic strength [23,25–26,30,35,38,81,86,91]. Moreover, an increase in divalent ions also leads to an increase in the formation of CDRF and decrease in LC concentration indicating a variable distribution of these photoproducts through intramolecular photoaddition and photoreduction, respectively [23,25–26,30,35]. The excited singlet state has been considered to be involved in the formation of CDRF and LC. However, the formation of LF through FMF has not been found to be much affected by an increase in buffer concentration which could be due to the involvement of the excited triplet state in the reaction. The same may also be hypothesized for LC as some of its fractions could be formed directly from FMF [21,23,25–26,30,35].

**Figure 7:**
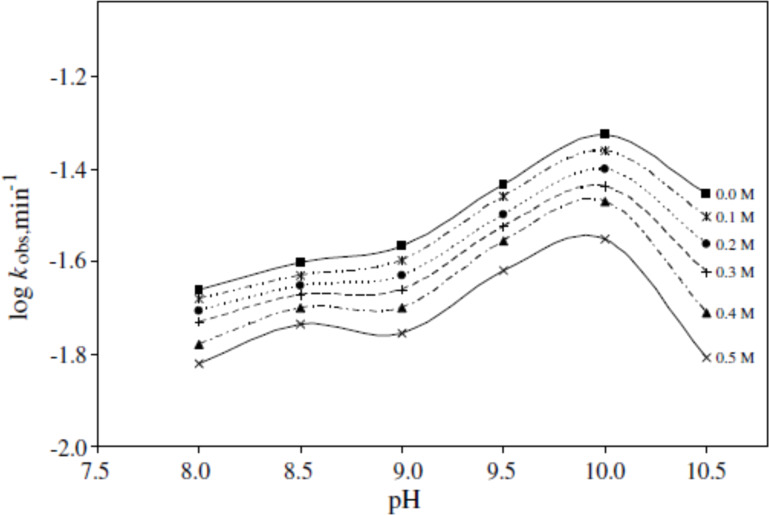
log *k*_obs_–pH profiles for the photolysis of RF (5 × 10^−5^ M) in 0.1–0.5 M borate buffer. Experimental conditions are the same as in Figure 6. Reproduced with permission from [28]. Copyright 2008 Elsevier.

On the contrary, some buffers such as borate and citrate have shown a stabilizing effect on the photolysis of RF [28,31]. One of such effects was observed in a dog when it was administered a solution of RF intravenously after its alkaline hydrolysis at room temperature (25 °C) for one hour. After administration, a drop in the blood pressure of the dog was observed. However, the same solution if immediately buffered with boric acid showed no such hypotensive activity even if the solution was kept for prolonged periods of time [77]. This stabilizing activity of borate ions was also observed by Wadke and Guttman [92], which was later confirmed by Ahmad et al. [28], who performed a kinetic study on the photolysis of RF in the presence of borate buffer at pH 8.0–10.5. It was found that with an increase in buffer concentration from 0.1 to 0.5 M, the rate of photolysis of RF slows down in a linear pattern following first-order kinetics (Figure 7).

**Figure 8:**
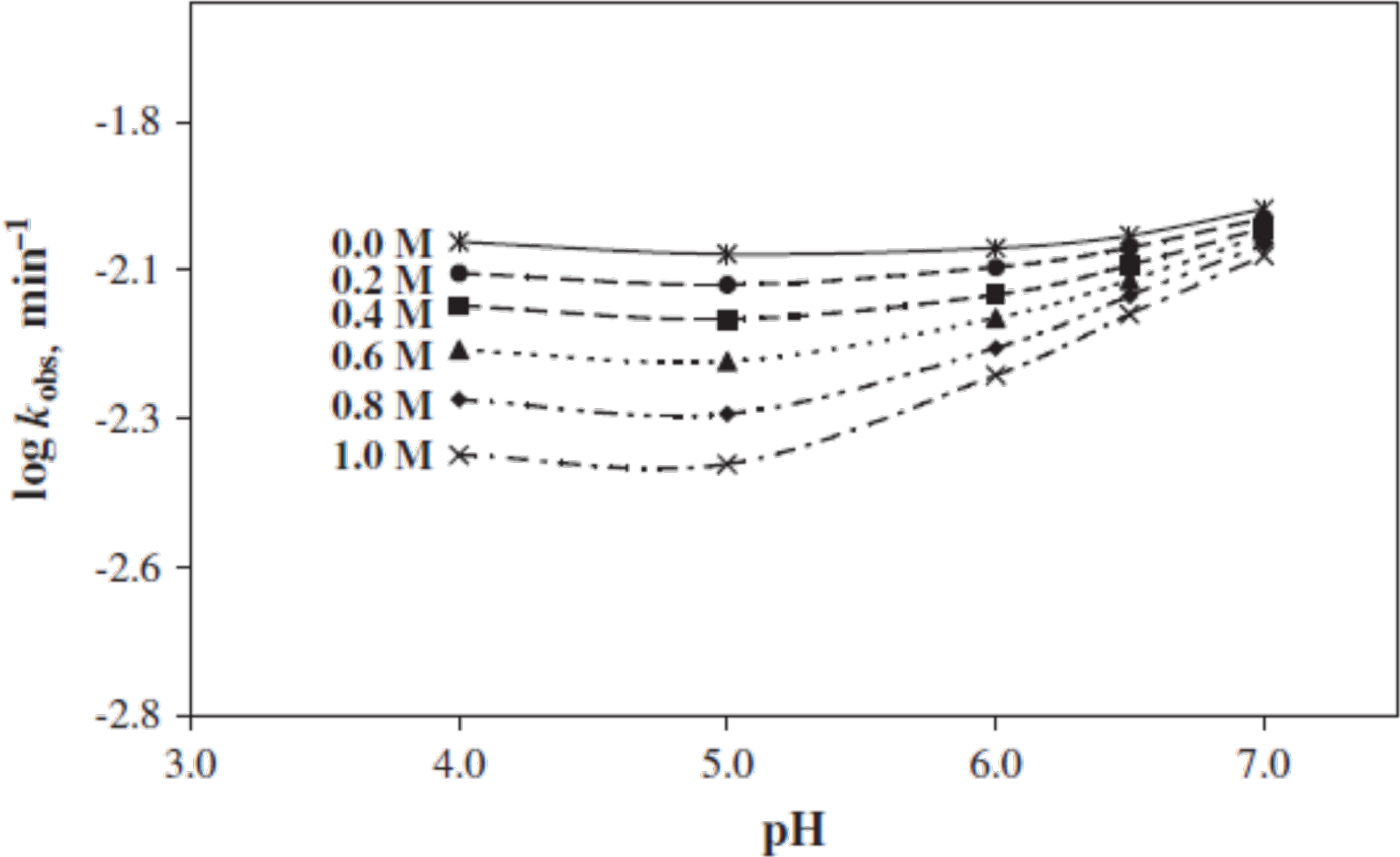
log *k*_obs_–pH profiles for the photolysis of RF (5 × 10^−5^ M) in 0.2–1.0 M citrate buffer. Experimental conditions are the same as in Figure 6. Reproduced with permission from [31]. Copyright 2011 Elsevier.

The inhibition of the photodegradation of RF by borate ions is due to the formation of a RF–borate complex involving the ribityl side chain [10,28,92–95]. Similarly, citrate buffer has also shown a stabilizing effect on the photolysis of RF solutions with increasing concentration (0.2–1.0 M) in the pH range of 4.0–7.0 (Figure 8). The trivalent citrate ions were found to have a greater inhibitory effect on the photolysis of RF as compared to the divalent citrate ions probably due to the quenching of the excited triplet state of RF [31].

#### Effect of solvent polarity and viscosity

The rate of RF photolysis is affected by solvent polarity, which causes changes in the conformation of the ribityl side chain to undergo degradation [83]. RF shows higher photostability in less polar solvents [96]. When RF was irradiated anaerobically in alcohols and alcohol/water mixtures, a slightly different photochemistry was observed which does not involve any primary photoreduction in the solvents. The major photodegradation products formed in alcohols were LC and FMF [83]. LC has also been identified as the major photoproduct of RF in various organic solvents such as acetic acid, acetone, dioxane and its mixtures with water, ethanol and pyridine [96–98]. The photodegradation of RF has been found to be more rapid in organic solvents as compared to aqueous solutions [97,99]. This could be linked to the physical properties of the solvents such as polarity, dielectric constant, viscosity, etc. [27,32,83,100–101]. The dielectric constant of the medium has been shown to affect complexation between RF and cloxacillin sodium in aqueous–ethanol media and found to decrease with an increase in temperature [102]. A 7–10% increase in the solubility of RF was observed when dissolved in methanol in the presence of various dendrimers [103]. A number of kinetic studies have been conducted on the photodegradation of RF and FMF in aqueous media at various pH values [17,23–35]. The quality of water also affects the rate of photodegradation of RF as it was found to be higher in D_2_O (66%) in comparison to that of distilled water (40%) [57]. The effects of various solvents on the rates of flavin redox reactions have been investigated using laser flash photolysis [50].

The effect of aqueous and organic solvents on the photolysis of FMF has been studied by employing a specific UV-visible spectrometric method [27,32,100–101]. The rates of photolysis of FMF were found to be different from that of RF as non-linear curves were obtained indicating that the photolysis of FMF does not follow first-order kinetics in water and organic solvents. The photolysis of FMF was found to be affected by the dielectric constants of the solvents, i.e., greater the dielectric constant higher the rate of photolysis. This indicated the involvement of a polar intermediate along the reaction pathway [50]. The values of the second-order rate constants for the aerobic and anaerobic photolysis of FMF with respect to solvent were found to be in the following order: water > acetonitrile > methanol > ethanol > 1-propanol > 1-butanol > dichloroethane > chloroform. The photoproducts formed on the irradiation of FMF in water included LC and LF as major and CMF as minor products. In the case of organic solvents, LC was the common major product in all solvents and CMF was the minor product in all cases except dichloromethane and chloroform [27,32]. Recently, a light-induced photolysis of four RF derivatives in methanolic solutions has also been reported and the products formed have been identified [85].

An attempt has been made to correlate the rate constant of anaerobic photolysis of FMF with solvent viscosity. A linear relationship has been observed between the second-order rate constants and inverse of solvent viscosity [32]. The dependence of flavin triplet state quenching on solvent viscosity has previously been reported [50]. A similar linear relationship between the rate constants and inverse of solvent viscosity has also been reported for ascorbic acid [104] and levofloxacin [105].

#### Effect of stabilizers, complexing agents and quenchers

Various methods have been considered to stabilize RF from photodegradation. These methods include the use of stabilizers, quenchers and complexing agents as discussed in the following sections.

**Stabilizers:** The effect of various stabilizers on the photostability of RF has been investigated by Asker and Habib [87]. They observed the greatest stabilizing effect by disodium ethylenediamine (EDTA) (96.2%), followed by thiourea (88.2%), methylparaben (86.4%), DL-methionine (76.3%), sodium thiosulfate (72.9%), ribonucleic acid (59.3%) and reduced glutathione (26.2%). When RF solutions were exposed to a 40 W fluorescent light (Sylvania fluorescent lamp with an intensity maintained at 1350 foot-candles), the photostabilizing effect of these agents was found to be dependent on their concentration as an increase in the effect was noted with an increase in concentration. Similarly, the pH of the medium and the buffer species (e.g., phosphate buffer), have been found to influence the rate of RF photodegradation in the presence and absence of EDTA [87]. The borate [28] and citrate [31] species have also been found to exert a stabilizing effect on the photodegradation of RF.

**Figure 9:**
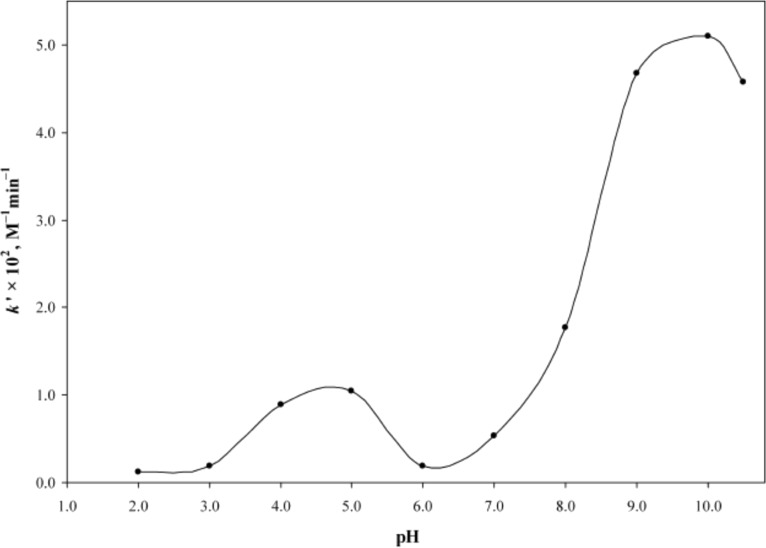
*k'*–pH profile for the photolysis of RF (5 × 10^−5^ M) in the presence of CF (0.5–2.5 × 10^−4^ M). Experimental conditions are the same as in Figure 6. Reproduced with permission from [29]. Copyright 2009 The Pharmaceutical Society of Japan.

**Complexing agents:** The use of various complexing agents is another way of RF photostabilization. Caffeine (CF) is known to form molecular complexes with RF [10,40,106–110] and thus slow down its rate of chemical [76] and photodegradation reactions [29,68]. A pH around 6 has been reported to be most suitable for the stabilization of RF in the presence of CF in pharmaceutical preparations (Figure 9) [29].

However, solutions containing both phosphate buffer and CF have been found to influence the photodegradation of RF by inhibiting the photoreduction pathway and enhancing the photoaddition pathway [35]. Different types of cyclodextrins have been studied for complexation with RF to achieve its stabilization [111–117]. In a comparative study of complexation between α- and β-cyclodextrins with RF, β-cyclodextrin was found to form more stable inclusion complexes with RF [116]. The formation of strong and stable inclusion complexes of RF with β- and γ-cyclodextrins have also been observed in other studies [111–115]. Such β-cyclodextrin complexes are suitable for fluorescent compounds for which the fluorescence intensity is influenced by the presence of cyclodextrins [113]. A non-inclusion complexation between RF and β-cyclodextrin or hydroxypropyl-β-cyclodextrin at low concentrations occurred through hydrogen bonding and resulted in a better solubility of RF along with an enhanced antitumor activity [117]. On the contrary, the formation of inclusion complexes between RF and hydroxypropylated α-, β-, and γ-cyclodextrins showed no stabilization effect towards RF. However, an enhancement in solubility was observed with hydroxypropylated β-cyclodextrin complexes [115]. Complexation between hydroxypropylated β-cyclodextrin and LC has also been reported which was found to be influenced by the pharmaceutical excipients such as vehicles (ethanol, propylene glycol), buffers (phosphate and citrate) and tonicity modifiers (NaCl, MgCl_2_) [118].

RF is also known to form complexes with dendrimers [103,119], certain drugs including antibiotics like cloxacillin sodium [102] and doxorubicin [120], dopamine [121], agents like *N*,*N*-dioctadecyl-[1,3,5]triazine-2,4,6-triamine [122], certain amino acids and indole [123–124], proteins [125] and metals such as Ag^+^, Ru^2+^ [126] to enhance the photostability of the vitamin.

**Quenchers:** RF on the absorption of light is promoted to the excited singlet state and then to the excited triplet state. These excited states eventually return to the ground state by emitting fluorescence, phosphorescence or heat. The falling back of these states to the ground state may be due to self-quenching of the RF molecule (internal quencher) or its photoproducts. Often external quenchers are added to RF preparations in order to alter the quantum yield of the photoreaction without quenching the fluorescence of RF [81]. Ascorbic acid and sodium azide are the two most studied external quenchers for RF photoreactions. Both these compounds reduce the photodegradation of RF with different quenching mechanisms. Ascorbic acid quenches both singlet oxygen and excited triplet states of RF whereas sodium azide quenches only the singlet oxygen in RF solution. Due to the dual activity, ascorbic acid is a comparatively better quencher than sodium azide. RF destructions were 94% and ~16% when photodegraded in the absence and presence of ascorbic acid, respectively [57]. Similarly, a 86% reduction in the formation of 2,3-butanedione was observed in RF solution when the concentration of sodium azide was increased from 0 to 5.0 mM [59]. A photochemical interaction between ascorbic acid and RF has also been studied in oil-in-water creams when irradiated with UV light [127]. Various other quenchers have been used to deactivate the excited states of RF such as β-carotene and lycopene [128], glutathione, D-mannitol [129], phenol [80], polyphenols such as catechin, epigallocatechin, and rutin [70], potassium iodide [81,129], purine derivatives such as uric acid, xanthine, hypoxanthine [130], α-, β-, γ- and δ-tocopherols [128], vitamin B_6_ family [131], xanthone derivatives [132], and 1,4-diazabicyclo[2,2,2]octane and 2,5-dimethylfuran [133].

#### Effect of formulation characteristics

Many considerations are given to the factors that are involved in the formulation of any dosage form. Such factors can affect the stability of the preparation and may result in the degradation of the active ingredient. The major factors related to RF photodegradation and photostabilization in solutions have already been discussed in the above sections. This section will particularly discuss the issues and factors related to the light mediated effects on solid dosage forms of RF including powders and tablets as reported by Sue-Chu et al. [73,134].

A color change in the powders and tablets containing RF as the active drug has been observed on exposure to light (xenon lamp, emission at 300–800 nm). The discoloration of samples was found to be affected by various factors such as the source of RF (i.e., synthetic or biosynthetic), occasional or continuous irradiation, tableting processes (wet granulation or direct compression), compression by means of IR press and excipients. On irradiation of the powder samples, the color change appeared instantly in the biosynthetic samples while gradually in the synthetic powder samples of RF at a radiation dose of ≤450 kJ/m^2^. After the rapid initial color change in biosynthetic samples not much change was observed whereas color continued to change in the synthetic samples and become more discolored upon continuous irradiation. An increase in color change in both powders was noted when the drug substance was compressed with an IR press at high pressure prior to exposure [73].

In the case of tablets, the two different forms of RF, i.e., synthetic and biosynthetic, showed an almost seven fold increase in discoloration indicating the catalyzing effect of excipients. The tablets formulated with synthetic RF powder, demonstrated the highest color changes in the presence of excipients such as icing sugar, lactose and wheat starch while those with biosynthetic RF, it was found to decolorize in the presence of nicotinamide, lactose, talc and sodium starch glycolate [73]. The color changes in solid RF are often reversible and are due to photochromism [73,134]. Such color changes might not affect RF quantitatively as the discoloration was only on the surface layer [73]. Moreover, a change of appearance does not always have a direct correlation with the chemical degradation and may, therefore, not affect the efficacy of the preparation [135–136]. However, such changes may end up in reducing the patient compliance [73].

#### Incorporation into liposomes

Entrapment of RF within lipid bilayered vesicles (liposomes) is another approach to improve the photostability of the vitamin and various studies related to such preparations have been conducted [111,137–143]. The composition of liposomes, pH of the preparation and concentration of ingredients may influence the photostability of the liposomal preparation as an increase in the concentration of dimyristoyl-phosphatidylcholine resulted in better photostability of RF. Similarly, an enhanced photostability of RF was observed in neutral or negatively charged liposomes while a decrease was noted in positively charged liposomes. The photodegradation of RF followed first-order kinetics both in the presence and absence of liposomes [138]. Highest stability of RF in a liposome was observed when the vitamin was complexed and entrapped in the aqueous phase. Moreover, the presence of at least one hydrophobic light absorber (e.g., oil red O) further improved its stability [111].

#### Effect of humidity

Photodegradation of aqueous solutions of RF has already been discussed in the previous sections. However, moisture can affect the stability of RF in dried form as acceleration in the photodegradation of RF in powder and tablets has been reported [73,134]. When the RF tablets were irradiated, a color change was noted immediately which was enhanced after 24 hours of storage in ambient conditions in dark. When similar samples were stored after irradiation in a sealed container with dried silica, no further modifications were observed in the samples. The analysis of the samples indicated the presence of loosely adsorbed moisture in the RF powder [134]. In a comparative study, the compressed mixtures of synthetic and biosynthetic RF powders and excipients were exposed to elevated humidity prior to irradiation. It was observed that the synthetic samples do not adsorb water even after 5 days of incubation whereas the biosynthetic samples adsorbed water after 24 hours of incubation. When the samples were exposed to humidity after irradiation, the results were quite different as most color changes appeared in the tablets with synthetic RF. Similarly, the tablets prepared through wet granulation showed maximum color changes as compared to those prepared by direct compression [73].

#### Effect of packaging material

Packaging material plays an important role in the photostability of RF. If RF is not packed in a suitable container even after storing at optimum conditions of pH, temperature, humidity, etc., it may degrade on exposure to light. A rapid loss of RF in milk has been reported in clear bottle or white sachet as compared to the milk packed in a brown bottle or carton [144]. Mestdagh et al. [145] performed a comparative study of RF photodegradation in milk by using four different types of polyethylene terephthalate (PET) packages. Their results indicated that the packages provided with additional light protection and triple white–black–white layers protected RF more efficiently from light exposure as compared to those with transparent appearance even if provided with a UV-absorbing additive. A similar type of RF photoprotection in milk and cheese has been reported by blocking all UV and visible excitation wavelengths by overwrapping the package [146] and storing the samples under colored filters [147] or using vacuum packaging [148]. Therefore, RF should always be stored in containers protected from air and light [1,73,149]. Alternatively, the tablets could be packed in unit dose containers or in the presence of a desiccant like dried silica to prevent moisture adsorption from the environment [73].

#### Thermal degradation of riboflavin

RF is a heat stable compound and little information is available regarding its thermal degradation in aqueous solution. However, some degradation pathways and products have been reported for the thermal destruction of RF [150–152].

Ahmad et al. [153] carried out a study of the thermal degradation of RF at 50–70 °C and identified a β-keto acid and a dioxo compound as the isoalloxazine ring cleavage products at pH 9–13. These authors developed a multicomponent spectrometric method for the simultaneous determination of RF and its thermal degradation products and evaluated the kinetics of degradation of RF and the formation of the two degradation products [154].

RF is a crystalline substance that melts in the range of 278–282 °C with decomposition [78,155]. It is stable to heat and is not affected by heating processes like hot air convection, infrared, high-pressure steam, or microwave during cooking [58] as well as to milk pasteurization [156]. Almost comparable first-order rate constants of 7.1 × 10^−3^, 7.0 × 10^−3^, and 6.6 × 10^−3^ min^−1^ were obtained for the thermal degradation of RF when whole green gram was cooked for 30 min in different ways such as in an open pan (t½ = 98 min), eco-cooker (t½ = 99 min) and pressure cooker (t½ = 105 min), respectively. Moreover, it was found that RF was comparatively more stable in green gram (t½ = 433–445 min from 50–120 °C) than in pure solution form (t½ = 408–419 min from 50–120 °C) after heating which could be due to the protective effects of the phytochemicals present in the green gram [157]. In an another study when chardonnay samples containing RF were irradiated, a rapid decrease in RF concentration was observed. When similar samples were kept in the dark and maintained at 45 °C, no change in RF concentration was noted over the studied time period indicating the thermal resistance of the vitamin [71].

The thermal degradation of RF is known to occur with a rise in temperature and exposure time [150,157–162]. When aqueous solutions of RF were heated for 40 min at 100, 120 and 150 °C, a degradation of 4, 7 and >20% was observed, respectively. Similarly, an increase of exposure time from 20 to 60 min at a constant temperature of 150 °C resulted in an enhanced thermal degradation of RF from approximately 15 to 42% [162]. In an another study, when soymilk was heated at 90–140 °C for 6 hours, the thermolysis of RF was found to follow first-order kinetics with the rate constants of 7.05 × 10^−4^, 4.26 × 10^−3^ and 2.12 × 10^−2^ min^−1^ at 90, 120 and 140 °C, respectively [159]. However, RF is thermally more stable to heat as compared to other vitamins such as thiamine and ascorbic acid [158–159]. The first-order degradation kinetics was also observed in thermally treated buffered solutions of RF at various pH values [150] as well as in its injections when exposed to light at elevated temperatures [160]. A general scheme for the thermal degradation of RF has been proposed by Mastowska and Malicka [151] which is based on TG, DTG and DTA analysis. The thermal degradation of RF initiates with its ribityl side chain by losing three molecules of water, followed by degradation to give a pyrrole ring, and formation of LC followed by its degradation. All these reactions take place at temperatures over 280 °C [151]. Similarly, the thermal behavior of RF complexed with certain metal ions such as Zn^2+^, Ni^2+^, Co^2+^, Cu^2+^, Ca^2+^, Mg^2+^ and Fe^3+^ has also been investigated [161]. It was observed that the most thermally stable complexes are formed with Zn and Ni, showing higher degradation temperatures as compared to pure RF whereas the remaining complexes showed similar or lower thermal stability to that of the pure RF with the Fe-complex found to be most rapidly degraded [161]. The presence of various metal ions, hydrochloric and sulfuric acid in aqueous RF solutions are also known to decrease the photodestruction rate of RF by 1.5–2.5 times. This could be due to protonation and formation of a complex between metal ions and oxygen atoms of hydroxy groups of RF [162].

Although RF is a thermostable substance, the temperature may greatly affect its stability if the pH of the medium is varied from the acidic to the alkaline region [11] or it is exposed to light [71,160]. Rapid destruction of RF in buffered solutions has been reported from pH 1.3–6.5 at 80 °C, pH 1.7–5.5 at 100 °C, pH 2.0–5.0 at 120 °C and below pH 1 and above pH 5.4 when heated at 121–123 °C for 1 hour [150–151]. Thermal studies have also been carried out on the degradation products of RF such as FMF and LF in order to better understand the reaction kinetics [79,163]. FMF when heated at 40–60 °C in acidic solutions in the dark undergoes thermal degradation by a second-order reaction and forms LC as the major product along with some minor side-chain products [163]. LF is another degradation product of RF which is formed in alkaline solution [46–47] and is unstable at elevated temperatures [79]. Urea and a quinoxaline carboxylic acid has also been reported as the thermal degradation products of RF in its aqueous solution [162].

#### Chemical degradation of riboflavin

Most of the studies carried out on the chemical degradation of RF involve the hydrolytic cleavage of the isoalloxazine ring in alkaline media. This leads to the formation of 1,2-dihydro-6,7-dimethyl-2-keto-1-D-ribityl-quinoxaline (flavo-violet, a dioxo compound) and a β-keto acid [75–77]. The formation of these products on the photolysis of RF at pH 10–12 by hydrolytic degradation of the isoalloxazine ring has been confirmed [24]. The base catalyzed degradation of 9–alloxazine, a RF analogue, has been studied at pH 9 and 13 and a number of products have been identified [93–94]. It has been suggested that the formation of the β-keto acid and the flavo-violet type compounds takes place through a 1,2-dihydro-1-methyl-2-oxo-quinoxaline-3-carboxyureide intermediate in the reaction [164]. The presence of similar compounds in the hydrolytic degradation of formylmethylflavin in alkaline media has been reported [33]. Earlier studies on the alkaline hydrolysis of FMF showed the formation of LC and LF by the cleavage of the formyl side chain [19,22]. Second-order rate constants for the formation of these products at pH 9–12 have been reported [22].

## Conclusion

RF as a vitamin participates in various biochemical reactions and is known to perform important biological functions. It takes part in electron transfer processes in biological redox reactions. RF is a highly photosensitive vitamin giving rise to several inactive products and needs a careful consideration of the factors affecting its stability. An optimum pH with most appropriate buffers would provide a better stabilization of the vitamin in aqueous solutions. Similarly, the addition of stabilizers, complexing agents, quenchers or incorporation into liposomes is also suggested for better protection of RF from photodegradation. Packaging of RF preparations in a suitable material which provides protection from light and humidity, along with storage at optimum temperature are also important for its stability. The thermal degradation of RF takes place at high temperatures and pH and does not occur under normal storage conditions, protected from light. RF and analogues are chemically degraded by cleavage of the isoalloxazine ring to produce a variety of compounds.
